# Type II and type IV topoisomerase mutations in clinical isolates of *Morganella morganii* harbouring the *qnr*D gene

**DOI:** 10.1186/s12941-014-0034-4

**Published:** 2014-08-09

**Authors:** Mariem Nasri Yaiche, Ikbel Denden Rafraf, Qinglan Guo, Maha Mastouri, Mahjoub Aouni, Minggui Wang

**Affiliations:** 1 Faculty of Sciences, Bizerta Carthage University, Bizerta, Tunisia; 2 Laboratory of Biologically Active Substances and Communicable Diseases (LR99ES27), Monastir University, Monastir, Tunisia; 3 Institute of Antibiotics, Huashan Hospital, Fudan University, Shanghai 200040, China; 4 Laboratory of Microbiology, Fattouma Bourguiba University Hospital, Monastir, Tunisia

**Keywords:** Morganella morganii, Quinolone resistance, Topoisomerase mutation, qnrD

## Abstract

**Introduction:**

The aim of this study was to show the emergence of the *qnrD* gene among fluoroquinolone-resistant *Morganella morganii* isolate. The occurrence of mutations in DNA gyrase (*gyrA* and *gyrB*) and topoisomerase IV (*parC*,*parE*) genes was also investigated in this strain.

**Methodology:**

95 clinical Enterobacteria were screened for harbouring the *qnrD* gene. The clinical isolate of *M. morganii* was recovered from urine from a patient hospitalized in the urology unit at Fattouma Bourguiba Hospital, Tunisia. Antibiotic susceptibility was tested with the agar disk diffusion method. Quinolone susceptibility was studied with microbroth dilution technique. The investigations of plasmid mediated quinolone resistance (PMQR) and topoisomerases mutations were performed by polymerase chain reaction and nucleotide sequencing.

**Results:**

This isolate showed high level of resistance to quinolones. The MIC with microbroth dilution technique was 512 μg/ml for norfloxacin, 256 μg/ml for ofloxacin and ciprofloxacin and 64μg/ml for levofloxacin.

This strain was found to harbour the quinolone resistance determinant *qnrD.* In addition, this strain harboured two new *gyrB* mutations (S463A, S464Y) and one *parC* mutation (S80I).

**Conclusions:**

This is the first report in Tunisia of *qnrD* determinant and tow new *gyrB* muations in *M. morganii*. The nosocomial infection due to this *proteeae* invites further study of its epidemiologic evolution.

## Introduction

The first *qnr* gene was observed in the late 1990s and described as a plasmid-mediated quinolone resistance gene (PMQR). Since that, five types of *qnr* genes have been reported: *qnrA*, *qnrB*, *qnrC*, *qnrD*, and *qnrS*. Their sequences are deposited at the following website (http://www.lahey.org/qnrStudies/). Most were observed in *Enterobacteriaceae*, located on large conjugative multi-resistant plasmids, such as pMG252 seen in the original *qnrA1*-positive strain [1].

The *qnrD* gene was first described in 2009 on a 4270 bp plasmid present in a human clinical isolate of *Salmonella enterica* serovar *Kentucky* and three *Salmonella enterica* serovar *Bovismordificans* isolates from China [2]. Since this report, *qnrD* was found in *Escherichia coli*[3], *M. morganii*[4], *P. Mirabilis*[4],[5], *Pseudomonas aeruginosa*[5] and *Providencia rettgeri*[6].

We describe the new *gyrB* and *parC* mutations in a clinical isolate of *M. morganii* harbouring the rare *qnrD gene that is first reported in Tunisia*.

## Material and methods

### Case presentation

On 22 November 2012, a 76-years old male presented to the urology department with hematuria and burning micturition that were neglected by the patient since six months. On presentation, he was not febrile with normal vital signs and negative urine culture.

The ultrasonography shows a bladder of budding aspect, bifocal with long axis (3 cm). The histopathology revealed urothelial carcinoma infiltrating high-grade bladder (II pTa). On 30/11/2012, underwent a transurethral resection of bladder tumor. A double-current probe 18 c was also introduced.

On 19/01/2013 the patient presented an acute pyelonephritis and the urine culture showed the presence of quinolone resistant *M. morganii.* The empiric antibiotic therapy consisted of three doses a day of 2_g Ampicillin during 4 days. Urine culture subsequently yielded *M.morganii*. The antibiotic regime was then replaced with three doses a day of cefoxitin 1_g during one week. After this, the urine culture was negative but the haematuria still existed.

### Bacterial strain

*M. morganii* was isolated from urine and identified using the Vitek 2 identification system (bioMérieux, Marcy l’Etoile, France). The antimicrobial susceptibilities of the *M. morganii* isolate were determined on Mueller-Hinton agar by the standard disk diffusion procedure as described by the Antibiogram Committee of the French Society for Microbiology (http://www.sfm.asso.fr). The microbroth dilution technique was used to determine the minimal inhibitory concentrations (MIC) for norfloxacin, ofloxacin, ciprofloxacin and levofloxacin.

### PMQR screening

The *qnrA, qnrB, qnrS, qnrC, qnrD, aac-Ib*, and *qepA* genes were detected by PCR with the primer sets as described previously [2],[7]-[10].

*QnrD* primers were used also for the clinical survey of *qnrD* gene in other quinolone resistance *Enterobacteriacae* (n = 95) collected from October 2010 to June 2013: *Klebsiella pneumoniae* (n = 59), *Enterobacter cloaceae* (n = 17), *Citrobacter freundii* (n = 9), *Salmonella spp* (n = 6), *Providencia rettgerii* (n = 3), *M. morganii* (n = 1).

### Conjugation

The transferability of *qnrD* gene between the *M. morganii* isolate and the *E. coli* J53Az^r^ recipient were carried out in LB broth. Cultures of donor and recipient cells in logarithmic phase (0.5 ml of each) were added to 4 ml of fresh LB broth and incubated overnight without shaking. Transconjugants were selected on Trypticase soy agar (TSA) plates containing sodium azide (100 μg/ml; Sigma Chemical Co., St. Louis, Mo.) for counterselection and gentamicin (10 μg/ml) to select for plasmid-encoded resistance.

### Detection of mutations in the topoisomerase II and IV genes

The quinolone resistance-determinant regions (QRDRs) of *gyrA*, *gyrB*, *ParC* and *parE* was amplified as described previously [11]. Products resulting from amplifications were subjected to sequencing. Analysis and comparison of nucleotide and amino acid sequence data were carried out using Lasergene software (version 7.1; DNASTAR, Wisconsin, USA), and programs available from the national Center for Biotechnology Information (http://www.ncbi.nlm.nih.gov).

## Results

### Antibiotic susceptibility of M. morganii

The clinical strain of *M. morganii* showed a low level of resistance to beta-lactams due to its resistance to ampicillin, amoxicillin, ticarcillin and piperacillin only. It was susceptible to amoxicillin/clavulanic acid, ticarcillin/clavulanic acid, piperacillin/tazobactam, cafalotin, cefamandol, cefoxitin, cefotaxim, ceftazidim, cefpirom, aztreonam, imipenem and ertapenem. This strain was resistant to gentamicin and tobramycin but susceptible to amikacin. It showed also, decreased susceptibility to trimethoprime/sulfamides and rifampicin. The MIC with microbroth dilution technique was 512 μg/ml for norfloxacin, 256 μg/ml for ofloxacin and ciprofloxacin and 64 μg/ml for levofloxacin.

### Detection of PMQR genes

The PMQR screening with PCR revealed the presence of *qnrD* in *M.morganii* only. None of the other studied Enterobacteria harboured the *qnrD* gene. The sequencing result showed the presence of *qnrD1* in the *M. morganii* isolate. *qnrA, qnrB, qnrS, qnrC, aac-Ib-cr* and *qepA* genes were not found in this strain.

### Conjugation

The conjugative reaction fails and the *qnrD* gene couldn’t be transferred to *E.coli* J53.

### Topoisomerase mutations

Sequencing of PCR products of QRDRs in *gyrB* and *parC* showed two mutations in the QRDR of *gyr B* at 463 and 464 codons. The first one is an S463A substitution and the second is an S464Y substitution.

Mutation was found also in the QRDR of *parC* at codon 80. It was an S80I substitution.

## Discussion

The Gram-negative *M. morganii* is found in the environment and as a part of the normal flora of humans. It has been considered as a rare cause of human infections. However, it is an important opportunistic pathogen in urinary tract, soft tissue infections and also in infections following surgery [12],[13]. In this case of study, the old age of the patient, the bladder tumor and its resection and the probe introduction after the surgery are risk factors leading to the urinary tract infection with the opportunistic *M. morganii*.

This *M. morganii* strain was highly resistant to quinolones and *qnrD* was detected. *qnrD* was not found to be self transmissible and it could have spread either on a mobilizable plasmid or on a transferable structure integrated into a conjugative plasmid [6]. The transfer of quinolone resistance due to *qnrD* is always studied on transformants never on transconjugants. In the study of Hu et al., *Proteus mirabilis* harbouring *qnrD* failed to transfer quinolone resistance to *E.coli* 600 by conjugation [14]. The MIC of ciprofloxacin in *E.coli* DH10B cells increased in the transformants carring the plasmid pBR322 with the cloned *qnrD* from 0,002 μg/ml to 0,06 μg/ml (by a factor 32) [2].

However, *qnrD* positive clinical strains described so far exhibited a high level of quinolone resistance with ciprofloxacin MIC ranging from 1 to higher then 32 μg/ml and they are likely to harbour other quinolone resistance mechanisms [14],[15]. Topoisomerase mutations have not been always investigated in *qnr*D positive clinical strains in order to evaluate how both mechanisms interact. The *gyrA* and *parE* primers used to amlify the QRDRs for Enterobacteria were not available for *M. morganii*. The study of Mazzariol A et *al.* couldn’t to amplify *gyrA* and *parC* genes in this bacteria and this is due to its unknown genome [15].

To date, none of *M. morganii* was described with *gyrB* mutations. In this report, we described for the first time, two new *gyrB* mutations in *M. morganii*. In gram-negative bacilli, *gyrB* mutations are rare in clinical strains and they have been described at positions 426, 431, 447, 463, 464 and 466. The Ser 463Ala mutation has been linked to *Klebsiella oxytoca*[11].

This is the first report of the previous mutation in *qnrD* harbouring *M. morganii*. The Ser 464 Phe mutation has been linked to quinolone resistant *S.enterica*, *P.aeruginosa* and *P.mirabilis*[16]-[18]. In this study, the *gyrB* Tyr464 mutation was different from those described previously.

For *M. morganii,* the first *ParC* mutation was reported in the Tunisian study of Mahrouki et *al.*[19]. It was an S80I mutation associated to the *qnrS* 1 gene.

Another *ParC* mutation (S80R) was also described previously in this *Proteeae*[20]. In this study, we described the first S80I mutation in qnrD-positive *M. morganii*. This type of mutation was already observed in other Enterobacteria but never associated to the *qnrD* gene [21].

In summary, we have reported for the first time in Tunisia the rare *qnr*D in *M. morganii* and also found two new *gyrB* mutations (S463A and S464Y) and one *parC* substitution (S80I). In addition to *qnrD* gene, both mutations in *gyrB* and *ParC* could contribute to the high level of resistance to quinolones in this strain. The nosocomial infection caused by this bacterium invites further study of its epidemiologic evolution.

## Competing interests

The authors declare that they have no competing interests.

## Authors’contributions

MNY carried the bacteria collection and typing antibiotic susceptibility, PCR screening, sequence blasting and writing the manuscript. IDR is project designer and co-supervisor of research work. QG Laboratory supervisor, she controlled the analysis, interpretation of data and controlled the manuscript. MM provided the necessary materials for collection, typing and doing the susceptibility tests. MA project designer and general supervisor of research group. MW General supervisor of research experiments and controlled the manuscript. All authors read and approved the final manuscript.

## References

[B1] Martínez-Martínez L Pascual A Jacoby GA Quinolone resistance from a transferable plasmid Lancet 1998 351 797 799 10.1016/S0140-6736(97)07322-4 9519952

[B2] Cavaco LM Hasman H Xia S Aarestrup FM QnrD, a novel gene conferring transferable quinolone resistance in *Salmonella enterica Serovar Kentucky* and *bovismorbificans* strains of human origin Antimicrob Agents Chemother 2009 58 603 608 10.1128/AAC.00997-08 19029321 PMC2630628

[B3] Zhao J Chen Z Chen S Deng Y Liu Y Tian W Huang X Wu C Sun Y Sun Y Zeng Z Liu J Prevalence and dissemination of oqxAB in *Escherichia coli* isolates from animals, farmworkers, and the environment Antimicrob Agents Chemother 2010 54 4219 4224 10.1128/AAC.00139-10 20696876 PMC2944610

[B4] Koncan R Mazzariol A Kocsis B Fontana R Lanzafame P Rassu M Cornaglia M First detection in Europe of plasmid-mediated fluoroquinolone resistance *qnrD* determinant Clin Microbiol Infect 2010 16 S126 10.1111/j.1469-0691.2009.02780.x

[B5] Ogbolu DO Daini OA Ogunledun A Alli AO Webber MA High levels of multidrug resistance in clinical isolates of Gram-negative pathogens from Nigeria Int J Antimicrob Agents 2011 37 62 66 10.1016/j.ijantimicag.2010.08.019 21074376

[B6] Guillard T Cambau E Neuwirth C Nenninger T Mbadi A Brasme L Vernet-Garnier V Bajolet O Champsa C Description of a 2,683-base-pair plasmid containing *qnrD* in two *Providencia rettgeri* isolates Antimicrob Agents Chemother 2011 56 1 565 568 10.1128/AAC.00081-11 21986831 PMC3256040

[B7] Cattoir V Poirel L Rotimi V Soussy C Nordmann P Multiplex PCR for detection of plasmid-mediated quinolone resistance *qnr* genes in ESBL-producing enterobacterial isolates J Antimicrobial Chemother 2007 60 394 397 10.1093/jac/dkm204 17561500

[B8] Minghua W Qinglan G Xiaogang X Xiaoying W Xinyu Y Shi W Hooper D Minggui W New plasmid-mediated Quinolone resistance gene, *qnrC*, found in a clinical isolate of *Proteus mirabilis* Antimicrob Agents Chemother 2009 53 5 1892 1897 10.1128/AAC.01400-08 19258263 PMC2681562

[B9] Park C Robicsek A Jacoby GA Sahm D Hooper D Prevalence in the United States of *aac(6*_*)*-*Ib*-*cr* encoding a Ciprofloxacin-modifying enzyme Antimicrob Agents Chemother 2006 50 11 3953 3955 10.1128/AAC.00915-06 16954321 PMC1635235

[B10] Yamane K Wachino J Suzuki S Arakawa Y Plasmid-mediated *qepA* gene among *Escherichia coli* clinical isolates from Japan Antimicrob Agents Chemother 2008 52 4 1564 1566 10.1128/AAC.01137-07 18285488 PMC2292543

[B11] Lascols C Robert J Cattoir V Bébéar C Cavallo J Podglajen I Ploy M Bonnet R Soussy C Cambau E Type II topoisomerase mutations in clinical isolates of *Enterobacter cloacae* and other enterobacterial species harbouring the *qnrA* gene Int J Antimicrob Agents 2007 29 402 409 10.1016/j.ijantimicag.2006.11.008 17254753

[B12] Tsai MT Yeh JT Yang WC Wu TH CAPD-related Peritonitis caused by *Morganella morganii* PD 2013 33 104 105 10.3747/pdi.2012.00035 PMC3598260 23349203

[B13] Chen Y Peng H Shia W Hsu F Ken C Tsao Y Chen C Liu C Hsieh M Chen H Tang C Ku T Whole-genome sequencing and identification of *Morganella morganii* KT pathogenicity-related genes BMC Genomics 2012 13 Suppl 7 S4 10.1186/1471-2164-13-S7-S4 PMC3521468 23282187

[B14] Hu Y Cai J Zhang R Zhou H Sun Q Chen G Emergence of *Proteus mirabilis* harboring *bla*KPC-2 and *qnrD* in a Chinese Hospital Antimicrob Agents Chemother 2012 56 5 2278 2282 10.1128/AAC.05519-11 22354308 PMC3346625

[B15] Mazzariol A Kocsis B Koncan R Kocsis E Lanzafame P Cornaglia G Description and plasmid characterization of *qnrD* determinants in Proteus mirabilis and *Morganella morganii* Clin Microbiol Infect 2012 18 E46 10.1111/j.1469-0691.2011.03728.x 22192340

[B16] Gensberg K Jin YF Piddock LJ A novel *gyrB* mutation in a fluoroquinolone-resistant clinical isolate of *Salmonella typhimurium* FEMS Microbiol Lett 1995 132 57 60 10.1111/j.1574-6968.1995.tb07810.x 7590165

[B17] Mouneimne H Robert J Jarlier V Cambau E Type II topoisomerase mutations in ciprofloxacin-resistant strains of *Pseudomonas aeruginosa* Antimicrob Agents Chemother 1999 43 62 66 9869566 10.1128/aac.43.1.62 PMC89021

[B18] Weigel LM Anderson GJ Tenover FC DNAgyrase and topoisomerase IV mutations associated with fluoroquinolone resistance in *Proteus mirabilis* Antimicrob Agents Chemother 2002 46 2582 2587 10.1128/AAC.46.8.2582-2587.2002 12121936 PMC127365

[B19] Mahrouki S Bourouis A Chihi H Ouertani R Ferjani M Moussa MB Barguellil F Belhadj O First characterisation of plasmid-mediated quinolone resistance-qnrS1 co-expressed *bla*_*CTX-M-15*_ and *bla*_*DHA-1*_ genes in clinical strain of *Morganella morganii* recovered from a Tunisian Intensive Care Unit Indian J Med Microbiol 2012 30 437 441 10.4103/0255-0857.103765 23183469

[B20] Andres P Lucero C Soler-Bistué A Guerriero L Albornoz E Tran T Zorreguieta A Galas M Corso A Tolmasky M Petroni A Differential distribution of plasmid-mediated Quinolone resistance genes in clinical enterobacteria with unusual phenotypes of Quinolone susceptibility from Argentina Antimicrob Agents Chemother 2013 57 6 2467 2475 10.1128/AAC.01615-12 23478955 PMC3716127

[B21] Lim S Lim K Lee H Jung S Kang M Nam H Prevalence and molecular characterization of Fluoroquinolone-resistant *Escherichia coli* isolated from diarrheic cattle in Korea J Vet Med Sci 2010 72 5 611 614 10.1292/jvms.08-0302 20009431

